# Age-Associated Epigenetic Alterations, Somatic Mutations, and Their Crosstalk in Alzheimer’s Disease

**DOI:** 10.1093/geroni/igab046.2410

**Published:** 2021-12-17

**Authors:** Yaroslav Markov, Kyra Thrush, Morgan Levine

**Affiliations:** Yale University, New Haven, Connecticut, United States

## Abstract

Aging is the major risk factor for Alzheimer’s Disease (AD), and as life expectancy increases, neurodegeneration will continue to afflict an ever-increasing proportion of the population. While numerous theories are attempting to explain the drivers behind AD pathology, what unites them is the observation that AD is reliably associated with a progressive buildup of age-related molecular changes. Because of the varying clinical presentations of AD in patients with similar genetic backgrounds, it has been postulated that epigenetics may be implicated in its etiology. Building on our prior work showing that AD pathology is linked to alterations in age-related DNA CpG methylation (DNAme) across various brain regions, we use state-of-the-art machine learning approaches to identify patterns of molecular damage in postmortem brain samples. We show that alterations in DNAme are associated with accelerated biological aging, AD, and the APOE e4 genotype, which is a major risk factor for AD. We also demonstrate that these associations are present in the PFC but not cerebellum -- in line with the current understanding of AD progression in the brain. Finally, we perform whole-exome sequencing and protein mass spectrometry on the same brain samples to test our hypothesis as to whether AD-associated alterations of DNAme are linked with the accumulation of somatic mutations that affect the structural and binding properties of protein epigenetic regulators.

